# The Rehabilitative Effect of Archery Exercise Intervention in Patients with Parkinson's Disease

**DOI:** 10.1155/2023/9175129

**Published:** 2023-06-08

**Authors:** Chiu-Ying Chen, Wei-Ning Wang, Ming-Kuei Lu, Yu-Wan Yang, Tsung Yu, Trong-Neng Wu, Chon-Haw Tsai

**Affiliations:** ^1^Graduate Institute of Clinical Medical Science, College of Medicine, China Medical University, Taichung, Taiwan; ^2^Department of Public Health, China Medical University, Taichung, Taiwan; ^3^Department of Nursing and Graduate Institute of Nursing, Asia University, Taichung, Taiwan; ^4^Institute of Population Health Sciences, National Health Research Institutes, Miaoli, Taiwan; ^5^Department of Neurology, China Medical University Hospital, Taichung, Taiwan; ^6^Ph.D. Program for Translational Medicine, College of Medicine, China Medical University, Taichung, Taiwan; ^7^Neuroscience and Brain Disease Center, China Medical University, Taichung, Taiwan; ^8^Department of Public Health, National Cheng Kung University, Tainan, Taiwan; ^9^Department of Healthcare Administration, Asia University, Taichung, Taiwan; ^10^School of Medicine, College of Medicine, China Medical University, Taichung, Taiwan

## Abstract

**Background:**

Archery exercise exerts a rehabilitative effect on patients with paraplegia and might potentially serve as complementary physiotherapy for patients with Parkinson's disease.

**Objective:**

This study aimed to examine the rehabilitative effects of an archery intervention.

**Methods:**

A randomized controlled trial of a 12-week intervention was performed in patients with idiopathic Parkinson's disease. Thirty-one of the 39 eligible patients recruited from a medical center in Taiwan participated in the trial, of whom 16 were in the experimental group practicing archery exercises and 15 were in the control group at the beginning; twenty-nine completed the whole process. The Purdue pegboard test (PPT), the Unified Parkinson's Disease Rating Scale I to III (UPDRS I to III), physical fitness test, and timed up and go test (TUG) were used to assess the intervention effects of archery exercise.

**Results:**

Compared to the control group, the outcome differences between the posthoc and baseline tests in PPT, UPDRS I to III, lower extremity muscular strength, and TUG in the experimental group (between-group difference in difference's mean: 2.07, 1.59, 1.36, −2.25, −3.81, −9.10, 3.57, and −1.51, respectively) did show positive changes and their effect sizes examined from Mann–Whitney *U* tests (*η*: 0.631, 0.544, 0.555, 0.372, 0.411, 0.470, 0.601, and 0.381, respectively; Ps < 0.05) were medium to large, indicating that the archery intervention exerted promising effects on improving hand flexibility and finger dexterity, activity functions in motor movement, lower extremity muscular strength, and gait and balance ability.

**Conclusions:**

Traditional archery exercise was suggested to have a rehabilitative effect for mild to moderate Parkinson's disease and could be a form of physiotherapy. Nevertheless, studies with larger sample sizes and extended intervention periods are needed to ascertain the long-term effects of archery exercise.

## 1. Introduction

Parkinson's disease (PD) is the second most prevalent neurodegenerative disorder after Alzheimer's disease, and its onset often occurs in the elderly. The estimated global burden of PD has increased from 2.5 million in 1990 to 6.1 million in 2016 and 9.4 million in 2020, and the age-standardized prevalence rates have increased by 21.7% [1, 2]. These changes have resulted in great public health concerns regarding PD prevention and control since society has aged worldwide. Taiwan faces the same challenge, as Taiwan has transformed into an aged society, and up to 40,000 patients have been diagnosed with PD [3]. The symptoms in patients with PD include motor symptoms such as bradykinesia, rigidity, static tremor, and postural instability, and nonmotor symptoms such as sleep disorders, cognitive impairment, anxiety, and depression. Without active treatment and intervention, these symptoms will become more severe as the disease progresses, eventually leading to a loss of daily living activities in patients with PD.

Motor symptoms affect the functional mobility, balance, and gait of patients with PD, which affect many activities of daily living. Pharmacological and neurosurgical treatment of PD may not exert optimal effects on reversing the deteriorating extremity function and mobility of patients with PD [4, 5]; moreover, long-term medication may lead to several motor complications [6].

The use of complementary and alternative medicine and physiotherapy has been recommended as adjuvant strategies to improve motor function, functional mobility, and balance. Tremor and impaired finger dexterity are recognized as early symptoms in patients with PD, and reductions in their amplitude and/or frequency are treatment targets. However, whether complementary approaches are beneficial for improving PD functions is still a highly debated issue [7], and along with medical interventions, exercises have been proposed as promising strategies [8]. Evidence on the effectiveness of a variety of exercises used to improve motor dexterity and reduce tremor has been provided, including treadmill exercise, cycling exercise, aerobic exercise, whole body vibration, stretching exercises, balance training, strength training, curved-walking training, and cued training [9–24]. Some types of exercises designed to enhance specific body functions, such as upper body karate training in a seated situation [25], tango exercises [26], hand movements using a virtual cube pick and place task [27], and limb pure eccentric training [28], were also reported to exert positive effects.

In addition to the usual exercise interventions described above, Qigong [29, 30], Tai Chi [31], Wuqinxi [32, 33], yoga [34], Irish set dancing [35], active theatre therapy [36, 37], and archery [15] were proposed as complementary therapeutic methods for improving the motor function of patients with PD, and empirical evidence showed more or less favorable effects not only on motor function but also on psychological well-being (e.g., the reduction in depression, apathy, and anxiety). Although archery training was much less applied, it appeared to be a promising approach for improving upper extremity functions.

Archery is a static sport with a stable sequence of movements throughout the shot. The movements include standing in a proper position with postural stability, pushing the bow, drawing the bow string with a three-finger hook, sighting the target, relaxing the flexor group muscles of the forearm, and actively contracting the extensors to release the arrow [38–40]. An archer should control relevant muscles to achieve a good shot; due to the nature of the archery, this exercise is assumed to have the potential to maintain or improve the extremity functions of patients with PD. However, very few intervention programs have applied archery in the rehabilitation of patients with PD and lack sufficient evidence on its effect; therefore, this study aimed to examine the effects of an archery intervention using a random-controlled trial.

## 2. Materials and Methods

### 2.1. Study Design and Participants

A randomized controlled trial of a 12-week archery training course was designed to improve the impairments associated with Parkinson's disease (PD). The clinical diagnosis of PD was based on the UK Brain Bank Criteria [41] and determined by neurologists with movement disorder expertise in China Medical University Hospital, Taichung, Taiwan. The criteria for eligibility were as follows: aged 40 years or older, on a stable medication regime, with a Hoehn and Yahr scale score ranging from 1 to 4, and no significant impairment in cognitive function assessed from the Chinese version of Cognitive Abilities Screening Instrument (CASI C-2.0) [42]. The exclusion criteria were as follows: unable to fully participate in the 12-week study period, already enrolled in another pharmaceutical or exercise intervention, extremity dysfunction severely affecting movement, frailty, severe impairments in vision, and any conditions considered by their neurologists unsafe and unsuitable to participate in the trial, for example, severe comorbidities such as diabetes and heart disease.

**Figure 1:**
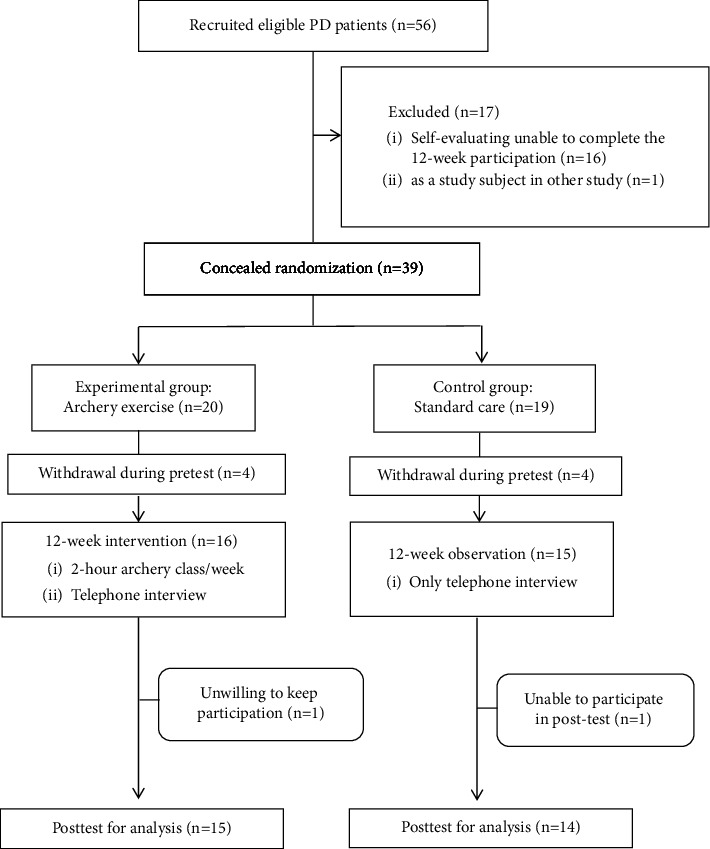
Flowchart of participants through the 12-week clinical trial.

After receiving approval from the Research Ethics Committee of China Medical University and Hospital (CRREC-106-083 AR-1), we started this study on December 7, 2017. Initially, 59 volunteers were recruited; and among the volunteers, 16 were unable to fully participate in the trial, and one already participated in another study. Therefore, 39 participants provided written informed consent, and a random assignment approach was then applied to allocate them into the experimental group (*n* = 20) and the control group (*n* = 19). During the pretest task for collecting baseline data, four of the participants in each group withdrew. During and after the 12-week course, one participant in the archery exercise intervention group withdrew and one participant in the control group was unable to complete the post-test task.  Figure 1 shows the flowchart of participant inclusion throughout the study period.

### 2.2. Intervention

The participants in the experimental group were assigned to take the archery training course. The course was designed by three archery trainers after discussion with the neurologists to provide participants with PD appropriate movements of drawing the bow and releasing the arrow. The trainers were traditional archery coaches with professional licenses issued by the Taiwan National Archery Association. This archery training aimed to improve the functions of the upper extremities; as patients with PD had stepping problems in terms of balance and stability, sitting postures were adopted for the participants' safety. Participants in the experimental group were assigned to two classes with approximately 10 participants in a class. Each trainer was assigned to instruct 3-4 participants who were all on medication and carefully directed each participant's performance on arching actions. Three-stage archery training was designed during the 12-week intervention. Basic arching (1st stage), advanced shooting (2nd stage), and integrative practice (3rd) were arranged in the first three weeks, the next four weeks, and the final five weeks, respectively. Each archery practice lasted for two hours and was held once a week in the Student Activity Center of the China Medical University next to its affiliated hospital. Before starting the archery practices, sitting posture exercises for warming up and stretching the upper extremities and finger skeletal muscles using elastic bands were led by coaches for approximately 15 minutes. For participant safety, a physical therapist was assigned to accompany them and observe their ongoing activities to avoid injury. Each participant was provided an elastic band and encouraged to practice the upper extremity and finger stretching actions at home to improve their archery skills. A telephone interview was conducted once a month to provide care and support to participants.

Participants in the control group received standard care. The care included a face-to-face personal interview educating them on how to stretch the upper extremities (UE) and finger skeletal muscles and self-care in daily living activities by delivering a pamphlet.

### 2.3. Outcome Measures

The primary outcome measure was tests of UE impairment and activity using the Purdue pegboard test (PPT). The test involves two abilities: gross movements of arms, hands, and fingers and fine motor extremity ability, through which finger dexterity and arm and hand coordination are assessed. Participants were asked to place as many pegs as they can and the number was recorded when time elapsed. The related information on PPT and its execution could be reached in elsewhere [43–45]. The secondary outcomes were several measures on assessing the progression of PD symptoms. Parts I to III of the Unified Parkinson's Disease Rating Scale (UPDRS) were used to evaluate the effects of the intervention. UPDRS consists of four parts, each of which consists of a 5-point ranking subscale. The first 4-item subscale is designed to assess intelligence, behavior, and emotions, including mentation, thought disorder, depression, and motivation/initiative. The second 13-item subscale is designed to assess daily living abilities, including speech, salivation, swallowing, handwriting, cutting ford, dressing, hygiene, turning in bed, falling, freezing, walking, tremor, and sensory symptoms. The third 27-item subscale is designed to assess motor ability, including speech; facial expression; resting tremor of the face, lips, chin, right hand, left hand, right foot, and left foot; action tremor of the hands (right and left); rigidity of the neck, upper extremities (right and left), and lower extremities (right and left); finger taps (right and left); hand grips (right and left); hand pronate/supinate (right and left); leg agility (right and left), rising from a chair; posture; gait; postural stability; and bradykinesia. The assessed score ranges from 0 to 4 points; the higher the score, the more severe the symptoms [46, 47]. Changes in body composition, limb muscular strength and endurance, limb flexibility, cardiorespiratory endurance, agility, and balance ability were also assessed using a physical fitness test suitable for the elderly. In addition, gait and balance were assessed using the timed up and go (TUG) test [48]. A logbook was delivered to participants to remind them to track medication records and the status of “on” and “off” drug effects.

### 2.4. Statistical Methods

The statistical analyses in this study were conducted using SPSS 25.0 for Windows. The chi-square and Fisher's exact tests were used to compare the discrete variables' distributions between the experimental and control groups; the Mann–Whitney *U* test was used to compare the continuous variables' distributions. The Wilcoxon signed-rank test and Mann–Whitney *U* test were used to examine the differences between the post-test and pretest in the outcome measures within and between groups, respectively. The effect size statistic was also provided, where a value less than 0.3 indicates a small effect, 0.3 to 0.5 indicates a medium effect, and greater than 0.5 indicates a large effect [49].

## 3. Results

At baseline (before implementing the archery intervention), equal distributions of sociodemographic characteristics and factors that may be associated with PD progression were observed between the experimental and control groups (Table 1). The mean age of patients with PD in the experimental group was 69.4 years (SD: 7.3), which was slightly older than that in the control group (67.2 years, SD: 10.7). More participants were male than female (over 50%), had at least a high school diploma, and most were married and in families with income levels ranging from 20,000 to 59,999 New Taiwan Dollar (NTD, symbol: NT$). The mean duration from the initial PD diagnosis was approximately 5 years; most patients were diagnosed with stage 2 PD, followed by stage 3. More than 60% reported a weekly exercise time >4 hours, and the drug that the patients took most was the levodopa. In addition, no differences in the UE impairments and activity functions at baseline were observed between the two groups, as shown in Table 2, in which the primary outcome (PPT) and the secondary outcomes (UPDRS, a physical fitness test for body composition, limb muscle strength and endurance, limb flexibility, cardiorespiratory endurance, and agility, and a TUG test for gait and balance abilities) were examined.

During the intervention, two subjects discontinued participation, and finally, 15 subjects in the experimental group and 14 subjects in the control group completed the post-test outcomes. The potential effects of this archery intervention on improving the physical functions of participants with PD are shown in Table 3, in which the comparison of the primary and secondary outcomes showed differences between baseline and posthoc tests, including differences within and between groups. The mean values of the differences between the post-test and baseline (post-test record minus pretest record) in the primary outcomes of right hand, left hand, and both hand PPTs within the experimental group indicated significant positive improvement (mean (SD): 1.77 (1.45), 1.06 (1.40), and 1.02 (1.19), respectively; Ps < 0.05), and the corresponding *η* values were greater than 0.5, showing large effects (*η*: 0.802, 0.609, 0.668, respectively). The mean values of the differences in the secondary outcomes, namely, UPDRS I to III, extremity flexibility (upper and lower), lower extremity muscular strength, cardio endurance, and TUG test, also showed significant positive improvements with large effects. All differences in outcomes between the post-test and baseline within the control group were insignificant, although most of the differences suggested that their corresponding functions tended to regress. Significant differences with medium to large effects in the differences between the post-test and baseline in the outcomes of PPT, UPDRS I to III, lower extremity muscle strength, and TUG tests between the experimental and control groups were observed (between-group difference's mean: 2.07, 1.59, 1.36, −2.25, −3.81, −9.10, 3.57, and −1.51 and effect size: 0.631, 0.544, 0.555, 0.372, 0.411, 0.470, 0.601, and 0.381, respectively; Ps < 0.05), suggesting that the archery intervention exerted promising effects on improving hand flexibility and finger dexterity, activity functions in motor movement, lower extremity muscular strength, and gait and balance abilities.

**Figure 2:**
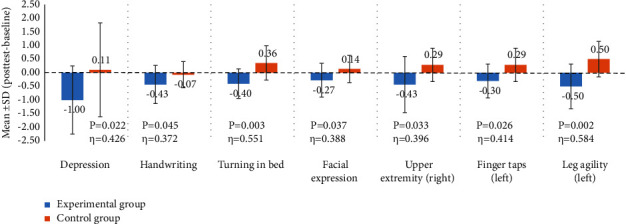
The significant differences in the within-group differences (post-test-baseline) for the assessed functions by UPDRS I to III between the experimental and control groups after the archery intervention.

The between-group differences in the outcomes between the post-test and baseline were analyzed using Mann–Whitney *U* tests to obtain more information on the effects of this archery intervention on each secondary outcome assessed using the UPDRS subscales (I to III). As shown in Figure 2, among UPDRS I items, the decreased depression score (mean ± SD: −1.00 ± 1.25) of the experimental group was significantly different (*P*=0.022) from the increased depression score (mean ± SD: 0.11 ± 1.72) of the control group, and the effect size was medium (*η* = 0.426). Among UPDRS II items, the severity in the function of handwriting was significantly decreased to a greater extent in the experimental group (mean ± SD: −0.43 ± 0.70) than in the control group (mean ± SD: −0.07 ± 0.48) (*P*=0.045), and the effect size was medium (*η* = 0.372); improvements in the functions of turning in bed and facial expressions were observed in the experimental group, but the functions in the control group were worse; both between-group differences were significant (mean ± SD: −0.40 ± 0.54 v.s. 0.36 ± 0.63, *P*=0.003; −0.27 ± 0.62 v.s. 0.14 ± 0.50, *P*=0.037, respectively), where the effect size for turning in bed was large (*η* = 0.551) and for facial expression was medium (*η* = 0.388). Among the UPDRS III items, the severity of right upper extremity, finger taps of the left hand, and left leg agility were significantly decreased in the experimental group but increased in the control group, and these between-group differences were significant (mean ± SD: −0.43 ± 1.03 v.s. 0.29 ± 0.61, *P*=0.033; −0.30 ± 0.62 v.s. 0.29 ± 0.61, *P*=0.026; −0.50 ± 0.82 v.s. 0.50 ± 0.65, *P*=0.002, respectively). The results also found a large effect for left leg agility (*η* = 0.584) and medium effects for the right upper extremity (*η* = 0.396) and for the left-hand finger taps (*η* = 0.414).

## 4. Discussion

During the archery intervention, no adverse events occurred, indicating that performing archery exercise while in a sitting posture is a safe intervention for patients with Parkinson's disease. Based on the results of the present study, archery exercise exerted a positive effect in improving several motor functions of patients with mild to moderate PD. One of the study strengths is the randomized controlled design and stratification of patients by PD disease stage (Hoehn and Yahr stages 1 to 4) to control for the confounding effects of unequal distribution of disease severity between the experimental and control groups on the intervention outcomes. The results in Tables 1 and 2 indicated comparable characteristics between groups before initiating the archery exercise course. Nevertheless, as most of the participants in this study were patients with stage 2 and 3 PD, the implication is that the effect of this archery rehabilitation intervention has limitations for patients with stage 4 PD and/or with severe symptoms.

Archery was one of the first sports introduced in the medical treatment of paraplegics and tetraplegics and was documented to be an ideal remedial exercise for training muscle groups of the arm, shoulder, and trunk muscles mainly used in archery [15]. To our knowledge, no rehabilitation program using archery exercises to help patients with PD has been developed; our study is the first to describe its effect by performing a randomized controlled trial.

The electrical activity of the muscle groups involved in the archery exercise was monitored using electromyography in an experimental study [15] and revealed that deltoids on both sides for securing horizontal and vertical positions of the arms, the biceps of the right arm for drawing and the left triceps for holding the bow in extension, the trapezius and rhomboid for bracing the shoulders while loading pulling the bow in horizontal draw, and latissimus dorsi and serratus anterior of the trunk muscles were all involved during the archery exercise. In addition, the palm of the hand, all finger muscles, and extensions of the wrist involved in the hook attachment and before the release of the arrow were exercised while performing the archery intervention. While a patient with PD focused on succeeding in shooting the target point from attaching the hook to the bow string, drawing the bow string back, twisting the hand, to releasing the arrow during the archery exercise, all involved muscle groups were activated and thus had the potential to enhance strength.

As patients with PD expressed many ambitions in pursuing success in shooting at the target point while performing the archery intervention in our study, this 12-week intervention of practicing archery actions along with using elastic bands to repeatedly activate their fingers, arm, trunk, and leg muscles helped patients with PD alleviate their symptoms, stabilize their gait and balance, and improve their extremity functions. These improvements were documented in the results of the present study, in which significantly greater differences in the post- and pretest numbers of placed nails (the changes of the primary outcomes of the three PPT tests) were observed between the experimental group and the control group (see Table 3), indicating that the archery intervention improves the functions of arms, hands, and finger dexterity in patients with PD. Significantly lower differences in post-and pretest scores of UPDRS I to III (the lower the value, the better the function) were observed for the experimental group than the control group (see Table 3), indicating that the archery intervention improved several motor functions required for daily living that mainly involve the upper and lower extremities, such as hand writing, turning in bed, facial expressions, right upper extremity, left finger taps, and left leg agility in particular (see Figure 2). Our findings are similar to the effects found in studies using other complementary therapies [29–37]. Additionally, participants in the experimental group improved their mentation, behavior, and mood (as assessed using UPDRS I) after the archery intervention, suggesting that practicing the archery exercise may have the potential to induce joyful feelings or even self-fulfillment feelings in patients with PD who succeeded in shooting the target point repeatedly, which in turn decreased their depressive symptoms. It is worth noting that similar to other complementary approaches such as Wuqinxi exercise [32, 33], yoga [34], and active theatre [36, 37], the archery approach also helps to improve psychological well-being.

The differences in outcomes indicated a significant increase in the lower extremity muscle strength, as assessed using physical fitness tests, and a significant decrease in the time required to complete the TUG test before and after the test in the experimental group compared to those in the control group (see Table 3), which also suggested that the archery intervention exerted a positive effect on increasing the muscle strength of the legs. A potential explanation for this finding is that practicing archery in a seated posture requires patients with PD to strengthen their lower extremity muscles to stabilize their upper extremities and trunk while they hold up the bow and arrows before releasing a shot. The strength of the muscles involved in performing a successful shot might be increased through archery exercises, which may in turn improve the gait and balance of patients with PD.

**Figure 3:**
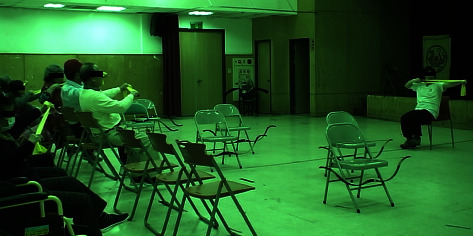
A training on drawing a bow by using an elastic band with the left leg raised for patients' practices at home.

**Figure 4:**
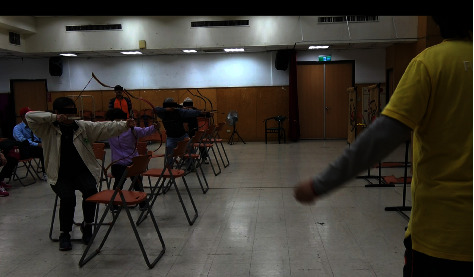
Patients performed archery with keeping their trunks a distance from the backs of their chairs.

In the archery intervention, the use of an elastic band at home by the PD participants to practice the actions involved in the archery classes may have a potential influence on the positive effects of this archery intervention.  Figure 3 shows an important part of the practice, in which a trainee was encouraged to practice the archery movement with one of the legs raised, the left leg in particular, by using an elastic band at home to stabilize their shooting actions for successfully reaching the target center in class.  Figure 4 shows the actual shooting practices in class. By performing these actions, the strength of the muscle groups involved in the action, namely, the arms, the right forearm, in particular, hands and fingers, was expected to be increased. The dexterity of the hands and fingers was also improved by the practice of buckling the string and gripping the bow with bent fingers using hand muscles. The aforementioned practices may explain the improved functions of the arms, hands, and finger dexterity assessed using the PPT and improved hand writing, decreased rigidity of the right upper extremity, and improved tapping function of the left finger assessed using UPDRS. In addition, a practicing archer with a seating posture that is asked to leave the body a distance from the back of the chair (see Figure 4) might also potentially improve the agility of the left leg and strengthen the trunk muscles. By stabilizing and balancing the whole body to achieve a good shot, the strength of the involved muscles would be enhanced by the aforementioned practices.

## 5. Conclusions

With the advantage of using a random clinical trial design, this study suggested that traditional archery exercise exerts positive effects on ameliorating a depressed mood and improving the motor functions of arms, hands, and legs and finger dexterity in patients with PD, such as hand writing, turning in bed, decreased rigidity of the right upper extremity, tapping movement of the left fingers, and agility of the left leg in particular. The strength of leg muscles and the gait and balance were also significantly improved. Nevertheless, due to the small number of eligible participants with PD and the short period of intervention (3 months) in this study, these conclusions should be interpreted with caution. Further studies using an RCT design that recruit more participants are needed to achieve sufficient statistical power, and the time of the intervention should be increased to ascertain the long-term effects of the traditional archery exercise.

## Figures and Tables

**Table 1:** The distribution of sociodemography and factors related to PD progression between the experimental and control groups at the baseline.

Outcome variables	Experimental group (*N* = 16)	Control group (*N* = 15)	M-W *U* (*Z*)/*χ*^2^ (*df*)	*P* value
	*N* (%)/mean (SD)	*N* (%)/mean (SD)		
Sociodemographic characteristics				
Age (years)	69.38 (7.25)	67.20 (10.73)	102.00 (−0.71)	0.476
Sex				
Male	9 (56.3)	11 (73.3)	0.99 (1)	0.320
Female	7 (43.7)	4 (26.7)		
Education				
Junior or less (<9 years)	5 (31.3)	7 (46.7)	0.78 (1)	0.379
Senior or more (≥9 years)	11 (68.7)	8 (53.3)		
Religion				
Yes	10 (62.5)	10 (66.7)	0.06 (1)	0.809
No	6 (37.5)	5 (33.3)		
Marital status^†^				
Single	1 (6.3)	1 (6.7)		0.654
Married	14 (87.5)	11 (73.3)		
Divorced	1 (6.3)	1 (6.7)		
Widowed	0 (0.0)	2 (13.3)		
Family income (NT$)^†^				
<20,000	2 (12.5)	2 (13.3)		0.142
20,000–39,999	3 (18.8)	7 (46.7)		
40,000–59,999	3 (18.8)	3 (20.0)		
≥60,000	8 (50.0)	2 (13.3)		
Missing	0 (0.0)	1 (6.7)		
PD disease status				
Time length (years)	4.65 (5.83)	5.47 (4.92)	90.00 (−1.19)	0.233
Hoehn and Yahr scale^†^				
Stage I	1 (6.3)	1 (6.7)		1.000
Stage II	11 (68.8)	10 (66.7)		
Stage III	3 (18.8)	3 (20.0)		
Stage IV	1 (6.3)	1 (6.7)		
Drug dosage taken (mg)				
Levodopa	372.66 (336.10)	370.00 (290.24)	113.00 (−0.28)	0.781
Benserazide	26.95 (26.98)	40.00 (49.43)	110.00 (−0.40)	0.687
Carbidopa	14.06 (35.32)	15.00 (42.05)	115.00 (−0.31)	0.758
Amantadine	59.38 (73.53)	23.33 (56.27)	83.50 (−1.69)	0.091
Pramipexole	0.12 (0.26)	0.11 (0.26)	119.00 (−0.06)	0.954
COMT	75.00 (204.94)	20.00 (77.46)	112.00 (−0.62)	0.538
Exercise^†^				
≤4 hours/week	6 (37.5)	4 (26.7)		0.704
>4 hours/week	10 (62.5)	11 (73.3)		

Note: ^†^denotes using fisher exact test; M-W *U*: the Mann–Whitney *U* statistic; *Z*: the *Z*-test score.

**Table 2:** The upper extremity impairments and activity functions between the experimental and control groups at the baseline.

Outcome variables	Experimental group (*N* = 16)	Control group (*N* = 15)	M-W *U* (*Z*)	*P* value
	Mean (SD)	Mean (SD)		
Purdue pegboard test				
Right hand test	10.57 (4.31)	10.20 (2.36)	95.50 (−0.97)	0.332
Left hand test	10.31 (4.10)	9.49 (2.95)	94.00 (−1.03)	0.303
Both hands test	8.04 (3.58)	7.40 (2.41)	97.00 (−0.91)	0.363
UPDRS				
UPDRS I	4.87 (3.42)	5.13 (3.23)	113.00 (−0.28)	0.781
UPDRS II	11.50 (7.76)	13.33 (8.42)	92.00 (−1.11)	0.267
UPDRS III	33.31 (18.90)	38.53 (17.20)	92.00 (−1.11)	0.268
Physical fitness				
BMI	24.64 (2.66)	24.89 (2.90)	110.00 (−0.40)	0.693
Waist-hip ratio	0.89 (0.08)	0.95 (0.18)	97.00 (−0.91)	0.363
UE flexibility	−19.63 (16.81)	−19.93 (13.61)	115.00 (−0.20)	0.843
LE flexibility	−6.44 (13.07)	−9.27 (11.50)	108.50 (−0.46)	0.649
UE muscle strength	15.19 (7.05)	16.87 (7.95)	99.00 (−0.83)	0.405
LE muscle strength	13.19 (5.04)	11.13 (3.80)	83.50 (−1.45)	0.147
Cardiorespiratory endurance	34.69 (14.64)	26.53 (12.87)	72.50 (−1.88)	0.060
Right side static balance	17.41 (13.62)	16.81 (13.05)	115.50 (−0.19)	0.850
Left side static balance	16.48 (12.79)	12.51 (10.23)	111.50 (−0.34)	0.735
TUG test	12.78 (7.64)	11.38 (4.34)	103.50 (−0.65)	0.514

Note: M-W *U*: the Mann–Whitney *U* statistic; *Z*: the *Z*-test score; UE: upper extremities; LE: lower extremities.

**Table 3:** The within-group and between-group differences in the outcome variables after the intervention period.

Outcome variables	Experimental group (*N* = 15)				Control group (*N* = 14)				Between groups (*N* = 29)		
	Post-test	Difference^(a)^	Effect size	*P* value	Post-test	Difference^(a)^	Effect size	*P* value	Difference^(b)^ in difference	Effect size	*P* value
	Mean (SD)	Mean (SD)	*η*		Mean (SD)	Mean (SD)	*η*		Z score	*η*	
PPT											
Right hand	13.04 (3.33)	1.77 (1.45)	0.802	0.002	9.99 (2.15)	−0.30 (1.11)	0.190	0.477	2.07 (−3.40)	0.631	0.001
Left hand	12.06 (3.22)	1.06 (1.40)	0.609	0.018	9.00 (2.95)	−0.53 (0.99)	0.477	0.074	1.59 (−2.93)	0.544	0.003
Both hands	9.60 (3.05)	1.02 (1.19)	0.668	0.010	7.11 (2.27)	−0.34 (0.97)	0.450	0.092	1.36 (−2.99)	0.555	0.003
UPDRS											
UPDRS I	2.47 (1.96)	−2.07 (2.71)	0.656	0.011	5.54 (2.90)	0.18 (2.89)	0.120	0.654	−2.22 (−2.01)	0.372	0.045
UPDRS II	6.97 (4.00)	−2.77 (3.37)	0.681	0.008	15.25 (9.09)	1.04 (3.87)	0.113	0.673	−3.81 (−2.21)	0.411	0.027
UPDRS III	23.80 (9.07)	−6.00 (9.92)	0.638	0.013	42.31 (14.87)	3.10 (8.01)	0.315	0.239	−9.10 (−2.53)	0.470	0.011
Physical fitness											
BMI	24.38 (2.74)	−0.09 (0.92)	0.081	0.755	24.65 (2.82)	−0.19 (0.37)	0.428	0.110	0.10 (−0.57)	0.105	0.570
Waist-hip ratio	0.89 (0.08)	0.01 (0.05)	0.138	0.594	0.95 (0.19)	−0.01 (0.07)	0.048	0.859	0.02 (−0.42)	0.077	0.678
UE flexibility	−12.50 (16.36)	5.10 (6.84)	0.617	0.017	−21.75 (20.70)	−1.46 (11.87)	0.014	0.959	6.56 (−1.75)	0.325	0.080
LE flexibility	3.07 (8.83)	8.00 (9.24)	0.714	0.006	−6.86 (10.84)	2.86 (6.82)	0.412	0.123	5.14 (−1.38)	0.256	0.168
UE muscle strength	15.67 (5.30)	0.27 (6.26)	0.037	0.887	13.29 (4.87)	−3.07 (5.73)	0.486	0.069	3.34 (−1.27)	0.237	0.203
LE muscle strength	18.00 (4.79)	4.14 (2.38)	0.824	0.001	11.57 (4.05)	0.57 (2.53)	0.137	0.609	3.57 (−3.24)	0.601	0.001
Cardio endurance	44.60 (9.34)	7.60 (8.24)	0.734	0.004	29.79 (12.31)	3.50 (6.16)	0.478	0.074	4.10 (−1.34)	0.248	0.182
Right static balancing	16.52 (13.14)	−2.05 (9.63)	0.107	0.678	13.71 (12.43)	−2.16 (5.76)	0.396	0.139	0.11 (−1.39)	0.242	0.193
Left static balancing	16.18 (12.31)	−1.39 (12.52)	0.162	0.530	12.35 (10.79)	−0.27 (2.92)	0.127	0.635	−1.12 (−1.48)	0.274	0.140
TUG test	9.90 (5.17)	−1.44 (1.04)	0.851	0.001	11.23 (5.18)	0.07 (2.11)	0.126	0.638	−1.51 (−2.05)	0.381	0.040

Note: (a) denotes the within-group difference = (post-test-baseline). (b) denotes the between-group difference = (the experimental-group difference's mean-the control-group difference's mean). *η* denotes the effect size obtained from dividing the absolute value of the *Z* score by squared *N*.

## Data Availability

The data are not available because based on Taiwan Personal Data Protection Act and Human Subjects Research Act, the study participants were assured that their data would remain confidential and would not be shared.
